# Prevalence of Hippocampal Sclerosis in a Clinicopathologically Characterized Cohort

**DOI:** 10.12988/cems.2013.13026

**Published:** 2013

**Authors:** Michael Malek-Ahmadi, Vickram Kahlon, Charles H. Adler, Aleksandra Obradov, Kabir Thind, Holly A. Shill, Lucia I. Sue, John N. Caviness, Sandra Jacobson, Marwan N. Sabbagh

**Affiliations:** 1 The Cleo Roberts Center for Clinical Research Sun Health Research Institute, Sun City, AZ, 85351, USA; 2 Parkinson's disease and Movement Disorders Center Mayo Clinic Scottsdale, Scottsdale, AZ, 85259, USA; 3 The Harold Civin Laboratory of Neuropathology Sun Health Research Institute, Sun City AZ 85351, USA; 4 University of Arizona, College of Medicine-Phoenix Campus Phoenix, AZ, USA

**Keywords:** hippocampal sclerosis, dementia, neuropathology, TDP-43, Alzheimer's disease, Parkinson's disease

## Abstract

**Background:**

Hippocampal sclerosis (HS) is a neuropathological finding that frequently occurs with pathologies, such as Alzheimer's disease (AD). Prevalence estimates of HS in autopsy-confirmed dementia samples have varied between 0.4% and 24.5%. However, the prevalence of HS within other pathologic groups has not been well characterized.

**Methods:**

Utilizing a sample of 910 prospectively followed and clinicopathologically confirmed dementia cases, we determined the prevalence of HS among the sample and within specific pathologic groups. HS prevalence of the sample was compared to reported HS prevalence rates in other autopsy-confirmed dementia samples.

**Results:**

The age range of the sample was 43 to 106 years, with a mean of 81.49±8.45. Of the 910 cases, 505 were male and 405 were female. For the entire sample, the average educational level was 14.59±2.65years. Of the 910 individuals, 47 (5.16%) cases had HS pathology present at autopsy. Among the 561 AD cases, 26 (4.43%) had HS pathology present. The frontotemporal dementia (FTD)/Pick's group had the highest percentage of cases with HS pathology (23.08%) followed by primary progressive aphasia (PPA) (16.67%) and Parkinson's disease with dementia (PDD) (5.34%). The HS prevalence rate of this study was not significantly different from all but 2 studies.

**Conclusion:**

The prevalence of HS pathology in this sample of autopsy-confirmed dementia cases was similar to other reported HS prevalence rates. This study is the first to report the presence of HS pathology in PDD cases.

## Introduction

Hippocampal sclerosis (HS) is a pathological finding of loss of neurons and subsequent gliosis in the CA1 section of the hippocampus and the adjacent subiculum [3]. HS pathology has been found concomitantly with Alzheimer's disease (AD), frontotemporal lobar degeneration with ubiquitin immunoreactive inclusions (FTDL-U), vascular dementia, and dementia with Lewy bodies [3,5] and is often localized and limited to the amygdala and entorhinal cortex [12]. Others have reported that HS may occur without the presence of other neuropathologies [9, 18, 28]. The etiology of HS has not been established, but vascular and excitotoxic hypotheses have been suggested [19]. HS pathology is associated with TAR-DNA (TDP-43) binding, however the physiologic mechanism behind this association has not been determined [25]. TDP-43 binding has also been observed in other pathologic groups [2, 13], so its presence is not specific to HS.

The reported prevalence of concomitant HS with dementia has varied with one study reporting a prevalence rate of 0.4% [1] while others have reported 9.5% [25] and 14% [26]. Corrada et al [10] report that among their cohort of individuals age 90 and above, 17% of autopsy-confirmed dementia cases had concomitant HS pathology. Beach et al [7] report a HS prevalence of approximately 10% in pathologically confirmed AD cases while HS prevalence was low among cases in which the primary pathologic finding was not AD (2/271). Hatanpaa et al [19] report on a pure form of HS which was found with no other primary pathological findings, but was not highly prevalent (1%) among their sample. Wilson et al [29] state that the prevalence of HS in cases with no antemortem clinical diagnosis is approximately 3%.

The current study was undertaken to compare the prevalence of HS among patients with different clinicopathologically confirmed dementias. Our aim was to determine the prevalence of HS pathology in a large, autopsy-confirmed dementia sample and to also determine the prevalence of HS pathology within several different clinicopathologic groups.

## Methods

### Subjects

Subjects were participants in the Brain and Body Donation Program at Banner Sun Health Research Institute in Sun City, AZ [6]. Participation and agreement for brain removal postmortem was voluntary and those with dementia at the time of agreement were signed in by their legal representative. Subjects also signed informed consent forms approved by the Sun Health Institutional Review Board signifying understanding of participation in this study. All subjects with clinically defined and pathologically confirmed dementia who came to autopsy between January, 1997 and December, 2011 were included in the study (N = 910).

#### Subject Classification

Parkinson's disease (PD) was diagnosed using the UK Brain Bank criteria [17], and Parkinson's disease dementia (PDD) was diagnosed using the criteria of Emre et al [15]. Alzheimer's disease was clinically diagnosed using the NINCDS-ADRDA criteria [21]. Neuropathological Alzheimer's disease cases were diagnosed using the criteria set forth by the National Institute on Aging/Reagan Institute, and categorized as either of “intermediate” or “high” probability [8]. Vascular dementia was diagnosed using criteria set forth by the NINDS-AIREN [28]. Frontotemporal dementia (FTD) and Pick's disease were collapsed into the same category and were diagnosed using the criteria set forth by the NFAV-PPA [24]. Dementia with Lewy bodies (DLB) and Alzheimer's disease Lewy body variants (AD-LBV) were also combined and were diagnosed using the criteria from McKeith et al [20].

#### Pathological Assessment

Neuropathological AD diagnoses were made according to National Institute on Aging/Reagan Institute criteria [8] and included those categorized as “intermediate” or “high” probability.

Senile plaque and neurofibrillary tangle load scores were obtained using the CERAD scoring system with separate semi-quantitative density estimates of none, sparse, moderate, or frequent (converted to a 0-3 scale for statistical purposes) using standardized published templates [22]. Regions scored included cortical gray matter from frontal (F), temporal (T), parietal (P), hippocampal CA1 (H), and entorhinal (E) regions. The individual carrying out the scoring (TB) was blinded to demographics and ApoE status.

HS was defined pathologically as virtually complete neuronal loss in the CA1 section of the hippocampus. In some cases, neuronal loss also was seen in the adjacent subiculum as a result of a lack of sufficient neurofibrillary tangles [18].

#### Statistical Analysis

Prevalence of HS for the study sample and each clinicopathologic subgroup was characterized by raw numbers and percentages. Pearson Chi-square was used to assess the degree of association between the presence of HS and gender among the study sample. A z-test for 2 proportions was carried out to determine if the prevalence of HS pathology in the current study was significantly different from that of other published HS prevalence rates. The z-scores -1.96 and +1.96 were used as the cutoff values to determine statistical significance as they correspond to a p-value of 0.05. Z-scores that were equal to or less than -1.96 and equal to or greater than 1.96 were used to indicate whether the prevalence rate of the current study was significantly different from that of others.

### Results

Data from 910 clinically diagnosed and pathologically confirmed dementia cases were analyzed. The age range of the sample was 43 to 106 years, with a mean of 81.49±8.45. Of the 910 cases, 505 were male and 405 were female. For the entire sample, the average educational level was 14.59±2.65years. Of the 910 individuals, 47 (5.16%) cases had HS pathology present at autopsy. HS pathology prevalence among the individual clinicopathologic groups is reported in Table 1. Chi-square analysis found no significant association of gender with HS (χ ^2^ = 0.08, df = 1, p = 0.78).

Among the 561 AD cases, 26 (4.43%) had HS pathology present. The FTD/Pick's group had the highest percentage of cases with HS pathology (23.08%) followed by PPA (16.67%) and PDD (5.34%). The progressive supranuclear palsy and posterior cortical atrophy groups had no cases in which HS pathology was present. HS pathology was present in 7.69% of vascular dementia cases and 5.88% of mixed dementia cases.

Table 2 displays the results for the z-test for two proportions which compares the HS prevalence rate of the current study to those reported previously. The prevalence rates of two studies were found to be significantly different from the current study. The prevalence rate reported by Ala et al [1] was significantly lower while the prevalence rate reported by Zarow et al [31] was significantly higher.

### Discussion

This study reports on the prevalence of HS pathology in a large, clinicopathologically confirmed cohort of dementia cases. The large sample allowed us to characterize HS prevalence not only among the sample, but also within specific pathologic groups. The prevalence of HS pathology in the entire sample was lower than some previously reported rates [4, 10, 11, 25, 26, 30], but was also higher than some [1, 7]. Ala et al [1] reported a prevalence rate that was significantly lower (0.4%), while Zarow et al [30] reported a prevalence rate that was significantly higher (24.5%). The prevalence of was not significantly different than that of several other studies [4, 7, 10, 11, 25, 26].

The relationship between HS and other neuropathological entities is unclear, however Nelson et al [25] report that HS neuropathology was not strongly associated with vascular pathology and that pure HS is an uncommon finding given the general prevalence of other age-related neuropathologies. One of the more interesting findings of this study is the presence of HS in PDD cases. Nakashima-Yasuda et al [23] report that TDP-43 binding was present in 7.2% of PD cases and 19% of PDD cases, while Beach et al [5] report that 7 of 12 cases with HS pathology demonstrated clinical parkinsonian symptoms. These studies suggest that HS pathology may co-occur with PD/PDD, however we are the first to report the pathological presence of HS pathology in PDD cases that were clinically diagnosed and pathologically confirmed.

Others have also reported on the relationship between HS and FTD [16, 19]. The current study did not directly assess this association, but did find that HS was present in approximately 23% of FTD/Pick's disease cases. This within-group prevalence rate was the highest among all of the pathologic groups. Lippa and Dickson [16] suggest that HS may fall within a spectrum of FTD-like diagnoses, but that a clear delineation between FTD and HS is difficult as HS pathogenesis may have multiple etiologies. However, Dickson et al [13] have suggested that a 3′ UTR variant on the progranulin gene, *GRN*, is associated with an increased risk of HS in older adults. In the context of previously established *GRN* mutation associations with FTD [27], these data suggest that FTD and HS share common genetic risk factors.

One strength of this study is the large number of clinically and pathologically confirmed dementia cases. This study was much larger than several others reporting the prevalence of HS among pathologically-confirmed dementia samples [4, 7, 10, 11, 26, 30] and was able to characterize HS prevalence in a variety of clinicopathologic entities. Others have conducted similar studies, however most have focused on the prevalence of HS with AD and FTD while our study provided HS prevalence rates among several other pathologic entities, including PDD.

One weakness of this study is the lack of a control group which could be used to more accurately characterize the association of HS among the different pathologic groups. The inclusion of individuals who were cognitively normal at time of death would help elucidate the associations between HS and other pathologic findings. A future area of study would be to characterize the prevalence of HS in cognitively normal older adults. HS in cognitively normal individuals has been investigated in the context of epilepsy studies which typically enroll younger adults and it has been suggested that these cases are pathologically different from HS associated with dementia in older adults [12].

## Figures and Tables

**Table 1:** Prevalence of Hippocampal Sclerosis by Pathological Diagnostic Group

Pathologic Group	HS+	HS–	Total	Within-Group HS Prevalence %	*Within-Sample HS Prevalence %
Alzheimer's Disease	26	561	587	4.43	2.86
AD-LBV/DLB	1	42	43	2.32	0.11
FTD/Pick's	6	20	26	23.08	0.70
Parkinson's Disease with Dementia	7	124	131	5.34	0.77
Vascular Dementia	2	24	26	7.69	0.22
Mixed Vascular Dementia	1	16	17	5.88	0.11
Posterior Cortical Atrophy	0	1	1	0.00	0.00
Primary Progressive Aphasia	1	5	6	16.67	0.11
Progressive Supranuclear Palsy	0	3	3	0.00	0.00
Dementia NOS	3	67	70	4.29	0.33
**Total**	**47**	**863**	**910**	**--------**	**5.16**

* = HS+ / 910

**Table 2:** Comparison of Published HS Prevalence Rates in Autopsy Cohorts

Study	Sample Size	HS Prevalence	z-score
Ala et al [1]	1771	0.4%	-2.36*
Barker et al [4]	382	13%	1.05
Beach et al [7]	154	1%	-0.91
Corrada et al [10]	104	17%	1.86
Dawe et al [11]	100	13%	1.32
Nelson et al [25]	1110	9.5%	0.58
Pao et al [26]	205	14%	1.30
Zarow et al [30]	130	24.5%	2.55*

* p<0.05; All comparisons are made against a sample size of 910 and prevalence rate of 5.16%.
